# Down Regulation of a Matrix Degrading Cysteine Protease Cathepsin L, by Acetaldehyde: Role of C/EBPα

**DOI:** 10.1371/journal.pone.0020768

**Published:** 2011-06-08

**Authors:** Riyaz A. Mir, Shyam S. Chauhan

**Affiliations:** Department of Biochemistry, All India Institute of Medical Sciences, Ansari Nagar, New Delhi, India; Kyushu Institute of Technology, Japan

## Abstract

**Background:**

The imbalance between extra cellular matrix (ECM) synthesis and degradation is critical aspect of various hepatic pathologies including alcohol induced liver fibrosis. This study was carried out to investigate the effect of acetaldehyde on expression of an extra cellular matrix degrading protease cathepsin L (CTSL) in HepG2 cells.

**Methodology and Results:**

We measured the enzymatic activity, protein and, mRNA levels of CTSL in acetaldehyde treated and untreated cells. The binding of CAAT enhancer binding protein α (C/EBP α) to CTSL promoter and its key role in the transcription from this promoter and conferring responsiveness to acetaldehyde was established by site directed mutagenesis, electrophoretic mobility shift assay (EMSA), chromatin immunoprecipitation (ChIP) assays and siRNA technology. Acetaldehyde treatment significantly decreased CTSL activity and protein levels in HepG2 cells. A similar decrease in the mRNA levels and promoter activity was also observed. This decrease by acetaldehyde was attributed to the fall in the liver enriched transcription factor C/EBP α levels and it's binding to the CTSL promoter. Mutagenesis of C/EBP α binding motifs revealed the key role of this factor in CTSL transcription as well as conferring responsiveness to acetaldehyde. The siRNA mediated silencing of the C/EBP α expression mimicked the effect of acetaldehyde on CTSL levels and its promoter activity. It also abolished the responsiveness of this promoter to acetaldehyde.

**Conclusion:**

Acetaldehyde down regulates the C/EBP α mediated CTSL expression in hepatic cell lines. The decreased expression of CTSL may at least in part contribute to ECM deposition in liver which is a hallmark of alcoholic liver fibrosis.

## Introduction

Alcohol induced hepatic fibrosis is preceded by the accumulation of ECM proteins in the liver. The accumulation of these proteins which are predominantly synthesized by the activated hepatic stellate cells is governed by the rate of their synthesis and degradation. A battery of proteases has been implicated in the degradation of these proteins. [1]–[3]. Many of these proteases are synthesized and secreted by both stellate cells and hepatocytes [4]–[6]. While the effect of alcohol or its oxidative metabolite acetaldehyde on the levels of various ECM degrading enzymes except CTSL have been extensively studied in stellate cells [7]. Very few studies on the effect of this metabolite on these enzymes have been carried out in hepatocytes [8].

CTSL, a lysosomal cysteine protease expressed by almost all types of eukaryotic cells is primarily involved in turnover and degradation of intracellular proteins [9], [10]. Over expression of this protease has been reported in a variety of human tumors and by malignantly transformed cultured cells [11], [12]. In cultured cells, after synthesis most of this protease is secreted in the medium. An intact carboxy terminal is essential for this process [13]. Secreted protease plays a key role in the invasion and metastasis of tumor cells due to its ability to degrade ECM over a wide range of pH [14], [15]. However, normal hepatocytes also synthesize and secrete large amounts of CTSL [16]. The secreted CTSL may play an important role in the homeostasis of ECM which is deposited in the fibrotic liver. However, no study delineating the role of acetaldehyde, an oxidative metabolite of ethanol in regulation of CTSL expression has been carried out in any cell type. Interestingly, Mantle et al [17], observed a complete loss of CTSL activity after *in vitro* treatment of human liver, brain and muscle tissues by acetaldehyde. These authors ruled out the role of direct inhibition of this protease by acetaldehyde but could not elucidate the molecular mechanism for the observed loss in enzymatic activity. Therefore, present study was planned to investigate the effect of acetaldehyde on the expression of CTSL, a potent ECM degrading protease and elucidate the molecular mechanism.

Our results demonstrate, for the first time transcriptional down regulation of CTSL expression in acetaldehyde treated HepG2 cells. The observed decrease in the expression of this protease which is mediated by the liver enriched transcription factor C/EBPα, may contribute at least in part to the accumulation of ECM proteins in alcoholics.

## Materials and Methods

### Reagents

Cbz-Phe-Arg-N-Methylcoumarin, cathepsin B inhibitor CA-074, Acetaldehyde, CTSL and α -tubulin antibodies were purchased from Sigma-Aldrich, St Louis, USA. The antibodies for C/EBP α lamin B, HRP-labeled and alkaline phosphatase-labeled mouse anti rabbit IgG were from Santa Cruz, CA, USA. Polyclonal antibodies for C/EBPβ and C/EBPδ were purchased from Abcam, Cambridge, UK. [*γ* -^32^P] ATP (3000 Ci/mmole) was procured from Board of Radiation and Isotope Technology, Jonaki, Hyderabad, India. `

### Cell culture and acetaldehyde treatment

HepG2 (Human hepatocellular carcinoma) cells were cultured as described earlier Arora and, Chauhan, [18]. Human hepatic stellate cells were obtained from Amal K. Santra, Centre for Liver Research, School of Digestive & Liver Diseases, Institute of Post Graduate Medical Education & Research, Kolkata, India and grown in DMEM F12-Ham media (Sigma – Aldrich, India) supplemented with 10% FBS (Gibco-BRL, Gaithusberg,MD, USA) and 20 ug/mL of Ciprofloxin. Acetaldehyde from a freshly prepared stock in PBS (300 µM/L) was added to the cells cultured in 25 Cm flask to a final concentration of 5 µM/L. After purging with 5% CO_2_ the flasks were capped tightly and wrapped in parafillm to prevent evaporation of acetaldehyde and incubated at 37°C for 24 hours. Growth rates of HepG2 cells in the presence and absence of acetaldehyde were assayed as described in by Xia et al. [19]

### Enzyme assay

Cells were washed twice with ice cold PBS and lysed in Tris HCl buffer (50 mM Tris HCl, pH 6.8; 150 mM NaCl; 10% Glycerol; 1% Nonidet P-40) followed by two freeze thaw cycles. The cell lysate was centrifuged at 10,000 g for 15 minutes at 4°C to remove the cell debris. The human CTSL (hCTSL) activity in the clear supernatant was assayed using a synthetic fluorogenic substrate, CBZ-Phe-Arg-N-Methylcoumarin in the presence of a specific cathepsin B inhibitor (CA-047) as described earlier [20].

### Western blotting

HepG2 cells were washed twice with ice cold PBS and lysed in 250 µl of RIPA buffer (50 mM Tris pH 7.4, 150 mM NaCl, 1% Nonidet P-40, 2 mM PMSF, 2.0 mM EDTA, 10.0 mM MgCl_2_) containing protease inhibitor cocktail (5 µg/ml) (Sigma, St Louis, MO, USA). The total proteins (50 µg) were resolved on SDS-PAGE (12%) and transferred on to a nitrocellulose membrane (Millipore, Bedford, MA). This membrane was incubated with 5% non fat dry milk powder in TBS (10 mM Tris–HCl, pH 7.4, 150 mM NaCl, 0.05% Tween-20) for 5.0 hours at room temperature to block the nonspecific binding. Then it was incubated with the primary antibody diluted in 1% non fat dry milk powder at 4°C overnight. Next day, immunoreactive proteins were detected with alkaline phosphatase conjugated or HRP conjugated secondary antibody using BCIP NBT substrate (Sigma-Aldrich, St Louis, USA) or Super Signal kits (Pierce Biotechnology, Rockford, IL) respectively as substrates according to the manufacturer's instructions.

### RNA isolation and Real time PCR

Total cellular RNA was isolated from the cultured cells was isolated using Trizole tri reagent (Sigma-Aldrich St Louis, MO, USA) according to the manufacturer's instructions. The integrity of the isolated RNA was checked on formaldehyde agarose gel. Real time PCR as described by Keerthivasan et al [20], was employed for the quantiation of CTSL, cathepsin B and cystatin C mRNA levels. The message levels have been expressed as a ratio of Ct values for these genes to 18S Ct values. The Ct values were averages from triplicate PCR reaction for each sample.

### Deletion analysis of C/EBP α binding motifs

The construction of various promoter reporter constructs used in the present study has been described in our earlier study [21]. We used the same PCR based strategy to sequentially delete the C/EBP α binding motifs from the hCTSL promoter. The nucleotide sequences of these primers used for making promoter –reporter construct containing one (pRB-7.5) and two (pRB-8.0) C/EBP α binding motifs are given in table-1.

**Table 1:** List of oligo nucleotides used for various purposes in the present study.

Oligonucleotides	Sequence	
**Cathepsin L**	**Sense:** 5′-GACTCTGAGGAATCCTATCCA-3′	180 bp amplicon
	**Antisense:** 5′-AAGGACTCATGACCTGCATCAA-3′	
**Cathepsin B**	**Sense:** 5′-TGTAATGGTGGCTATCCTGCT-3′	180 bp amplicon
	**Antisense:** 5′-AGGCTCACAGATCTTGCTACA-3′	
**Cystatin-C**	**Sense:** 5′-CAGCAACGACATGTACCACAG-3′	180 bp amplicon
	**Antisense:** 5′-TTCCTTTTCAGATGTGGCTGGT-3′	
**EMSA oligos**	**C/EBP.1 WT Sense:** 5′- TTTTT**ATG**TAATAA -3′	
	**C/EBP. 1 WT Antisense:** 5′- TTATTA**CAT**AAAAA -3′	
	**C/EBP.2 WT Sense:** 5′-TAATTA**CAT**AAATT-3′	
	**C/EBP. 2 WT Antisense:** 5-′AATTTAT**GTA**ATTA-3′	
	**C/EBP.1 MT Sense:** 5′- TTTTT**tct**TAATAA -3′	
	**C/EBP.1 MT Antisense:** 5′- TTATTA**aga**AAAAA -3′	
	**C/EBP.2 MT Sense:** 5′- TAATTA**aga**AAATT-3′	
	**C/EBP.1 MT Antisense:** 5-′AATTTAT**tct**ATTA-3′	
**ChIP primers** Flanking the region of cathepsin L promoter containing C/EBP alpha motifs	**Sense: CF** 5′-TGGGGTAAAGGCAGAGGTAA-3′	198 bp amplicon of mRNA
	**Antisense: CR** 5′-TGGAGAAATGTTGTAAGAGGAAA-3′	
**C/EBPα siRNA 1 sequences** cloned in pSilencer 1.U6 siRNA expression vector having Apa *I* and Eco R*I* flanking sites	**Sense1: ** 5′-TCTAGTATTTAGGATAACTTCAAGAGAGTTATCCTAAATACTA G AGTTTTTG-3′	Target region 1378–1392 of mRNA
	**Antisense 1:** 5′-**AATT**CAAAAACTCTAGTATTTAGGATAA CTCTCTTGAAGTTATCCTAAATACTAGAG**GGCC**-3	Apa *I* and Eco R*I* adaptor sequences have been shown in **bold face**
**C/EBPα siRNA 1 sequences** cloned in pSilencer 1.U6 siRNA expression vector having Apa *I* and Eco R*I* flanking sites	**Sense 2:** 5′-CGGTTGTTCCCCTAGTTCTATTCAAGAGATA GAACTAGGGGAACAACCTTTTTG-3′	Target region 1621–1639 of mRNA
	**Antisense 2:** 5′-**AATT**CAAAAAGGTTGTTCCCCTAGTTCT ATCTCTTGAATAGAACTAGGGGAACAACCG**GGCC**-3′	Apa *I* and Eco R*I* adaptor sequences have been shown in **bold face**
**Scrambled siRNA sequences** cloned in pSilencer 1.U6 siRNA expression vector having Apa *I* and Eco R*I* flanking sites	**Sense:** 5′-CTTAAGTCATAGCTATTGAATTCAAGAGAT TCAATAGCTATGACTTAAG-3′	
	**Antisense: ** 5′-**AATT**CTTAAGTCATAGCTATTGAATCTC TTGAATTCAATAGCTATGACTTAAG**GGCC**-3′	Apa *I* and Eco R*I* adaptor sequences have been shown in **bold face**
**Oligos used for deletion construct of CTSL promoter**	**pRB8 Sense:** 5-**GGTACC**AAGGTTATGGGGTAAAGGCA GAGG-3′	Kpn*I* site (**Bold face**)
	**pRB7.5 Sense:** 5′CGG**GGTACC**GAAATGACAAAAGTT TAGAATG-3′	
	**Antisense:** 5′-AATT**AAGCTT**TCCTGCTGCGGTCGTAGC TGC-3′	Hind*III* site (**Bold face**)
**Oligos used for site directed mutagenesis**	**pRB9 Sense:** 5′-AAGGTACCAGCCTGAACAACAGAGCC A-3′	
	**C/EBP 1 Mut Antisense:** 5′-GTCATTTCATTTTTTATTA**aga**AAAA ACATTCATTG-3	
	**C/EBP 1 Mut Sense:** 5′-CTACAATGAATGTTTTT**tct**TAATAAAA AAT GAAATG-3′	
	**C/EBP 2 mut Sense:** 5′-ATCCTTAATTA**aga**AAATTAAT ATCTACA-3′	
	**C/EBP 2 mut Antisense: ** 5′-GTAGATATTAATTT**tct**TAATTAAGGAT-3′	
	**RSBI Antisense:** 5′-AATTAAGCTTTCCTGCTGCGGTCGTAGCTGC-3′	

**Mutated nucleotides are shown in lower case and bold face.**

### Mutagenesis of the C/EBPα binding motifs in hCTSL promoter

The C/EBPα binding motifs at −1112/−1126 (TTTTTATGTAATAA) and −1044/−1058 (TAATTACATAAATT) positions of hCTSL promoter were mutated to TTTTT**tct**TAATAA and TAATTA**aga**AAATT respectively. The mutated nucleotides have been shown in lower case. The mutagenesis was essentially carried out as described by Antona et al [22] 2003. The primers used are given in table-1.

### Cell transfections and luciferase assay

HepG2 cells (1×10^5^) were seeded in each well of a six well plate one day prior to the transfection. Next day cells were washed with cold serum free DMEM and transfected with 1.0 µg of the promoter reporter construct and 25 ng of pRL null vector (Promega, Madison, WI, USA) using Transfast ™(Promega)according to manufacturers protocol.12.00 hours after transfection the cells were harvested and processed for luciferase assay as described earlier [20]. The luciferase activity was expressed as LU/s.

### Nuclear extract preparation

Cells were washed twice with ice cold PBS and collected in a 15 ml polypropylene tube with a rubber policeman and gently pelleted. Then cells in the pellet were lysed in sucrose buffer (Sucrose 0.32 M, Tris-HCl 10.0 mM (pH 8.0), MgCl_2_ 3.0 mM, Magnesium-acetate 2 mM, EDTA 0.1 mM and NP-40 0.5%) and centrifuged at 500×g for 5 minutes. The nuclear pellet was again washed with sucrose buffer without NP-40 and resuspended in low salt buffer (HEPES 20 mM (pH 7.4), Mgcl_2_1.5 mM, KCl 20 mM, EDTA 0.2 mM 25% glycerol and 5 ul protease inhibitor/ml (Sigma). The nuclear suspension was mixed with an equal volume of high salt buffer (20 mM HEPES pH 7.4, 1.5 mM Mgcl_2_, 800 mM KCl, 0.2 mM EDTA 25% glycerol and 5 µl/ml protease inhibitor) the nuclear suspension was incubated on ice for 45 minutes to release the nuclear proteins. The soluble nuclear proteins were separated from the insoluble nuclear fraction by centrifugation at 1400×g for 5 minutes. After assaying total proteins by BCA kit (Pierce Thermo Scientific) the soluble nuclear fraction was stored at −80°C in small aliquots till further used.

### Electrophoretic mobility shift assay

Complementary oligonucleotides (sequences given in table-1) encompassing (−1112/−1126) or (−1044/−1058) C/EBPα binding motifs of hCTSL promoter were annealed and end labeled with γ ^32^P-ATP using T-4 polynucleotide kinase (New England Biolabs,) according to manufacturers instructions. The free radioactivity from the radiolabeled DNA fragments was removed by G-25 column chromatography before using them for EMSA. The EMSA was performed using Promega's Gel Shift Assay System according to manufacturer's protocol. All the binding reactions were carried out at room temperature for 20 min using 5.0 µg of HepG2 nuclear lysate and 5.0 pmol of radiolabeled probe in buffer containing 2% glycerol, 0.5 mM MgCl2, 0.25 mM EDTA, 0.25 mM DTT, 25 mM NaCl, 5 mM Tris-HCL pH 7.5, 0.025 mg/ml poly (dI-dC)-(dI-dC). An excess 100 molar excess of unlabelled wild type or mutated probe was added to the binding reactions for specific and non specific competitive assays. For supershift assays 8.0 µg of the specific antibody against C/EBPα was added to the binding reactions followed by overnight incubation at 4°C. The protein-DNA complexes were resolved on 6% non-denaturing PAGE in 0.5× TBE buffer and visualized by autoradiography.

### Chromatin immunoprecipitation(ChIP)

ChIP assays were performed using Imprint™ Chromatin Immunoprecipitation Kit (Sigma-Aldrich, St. Louis, USA) according to the manufacturer's protocol. 2.0 µg of antibody, diluted in the 100 µl antibody buffer was incubated for 90 mins in the strip wells provided in the kit. Simultaneously, 1×10^6^ HepG2 cells were treated with 1% formaldehyde for 10 min at 25°C to cross link the existing DNA-protein complex(s). After treating with glycine (125.00 mM) to quench crosslinking, the cells were processed for the isolation of nuclei. The nuclear pellet was resuspended in the shearing buffer provided in the kit and subjected to sonication using a Misonix sonicator at a power setting of 1.5 and a 100% duty cycle, the extracts were sonicated for three 10-s pulses, with two minutes on ice in between pulses. The sheared chromatin was separated from the cell debris by centrifugation at 14,000 rpm for 10 min at 4°C. The supernatant was incubated in the wells pre-coated with the antibody for 90 mins. The immunoprecipitated DNA was recovered and used as template for PCR using CF and CR as sense and antisense primers complementary to the region flanking the C/EBPα motifs on hCTSL promoter. The nucleotide sequences of these primers are given in table-1. The products were resolved on agarose gel, purified and sequenced. PCR was performed with an aliquot of sheared chromatin DNA before immuno-precipitation and served as input control.

### Construction of C/EBPα si RNA expression vector

Ambion siRNA Target Finder, an online tool was used for predicting the siRNA sequence against C/EBPα. The complementary oligonucletides containing the predicted siRNA sequences were annealed, phosphorylated and cloned in expression vector pSilencer 1.-U6 (Ambion, Austin, TX, USA). In this way two human CEBP α siRNA expression vectors namely pU6/C/EBPα -1 and pU6/C/EBPα-2 were constructed. Similarly a scrambled siRNA expression vector (pU6/SC) was also generated which served as negative control. The sequences of oligonucleotides used for construction of these vectors are given in table-1. To determine their silencing efficiencies these vectors were transiently transfected in HepG2 cells and the expression of C/EBPα was assessed by Western blotting.

### Statistical analysis

The results in the present were analyzed using paired two tailed student's-t test. p value of ≤0.05 was considered statistically significant.

## Results

### Acetaldehyde treatment decreases CTSL expression in HepG2 cells

**Figure 1:**
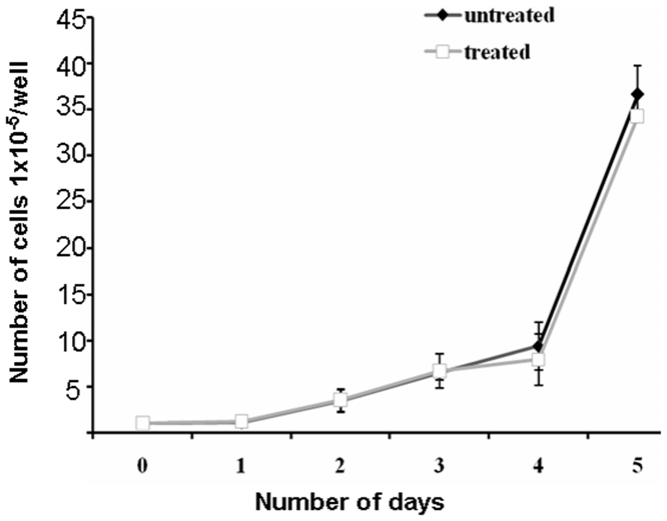
Growth rates of HepG2 cells in the presence and absence of acetaldehyde. 10^5^ cells were plated in each well of a six well plate and allowed to grow in the presence or absence of 5.0 µM acetaldehyde. After various time points the cells were trypsinized and counted. Values are mean ±SD of three independent experiments performed in triplicate.

**Figure 2:**
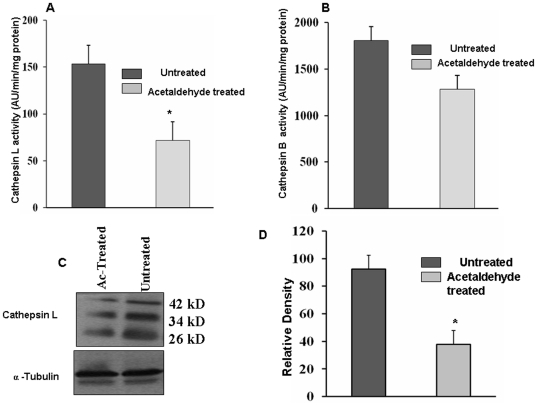
Reduction in the activity and levels of CTSL by acetaldehyde treatment in HepG2 cells. Cathepsin L+cathepsin B activity was assayed in the cell lysates spectroflourimetrically as described in materials and methods. The specific activity of CTSL was measured by including a specific cathepsin B inhibitor (CA-074) in the assay mixture and used to calculate cathepsin B activity, by subtracting it from the total enzymatic activity of L+B. (A) Effect of acetaldehyde treatment on CTSL activity (B) Effect of acetaldehyde treatment on cathepsin B activity. Values are mean ± S.D from at least three independent experiments performed in triplicate. Values significantly different from untreated controls have been marked by *(P≤0.05). (C) Effect of acetaldehyde treatment on CTSL levels. CTSL levels in acetaldehyde treated and untreated cells were detected by western blotting using a monoclonal antibody. Simultaneously western blot for α Tubulin was performed. Representative blots are shown in figure. (D) Densitometric quantitation of CTSL levels in acetaldehyde treated and untreated cells. The three specific bands of CTSL (42 kDa, 34 kDa and 26 kDa) representing prepro, pro and mature forms of the protease were quantitated densitometrically and the values thus obtained for each form were added. The values obtained for α-tubulin were used as an internal control for equal loading. Values are mean ± S.D from three independent experiments. Values significantly different from untreated controls have been marked by *(P≤0.05).

**Figure 3:**
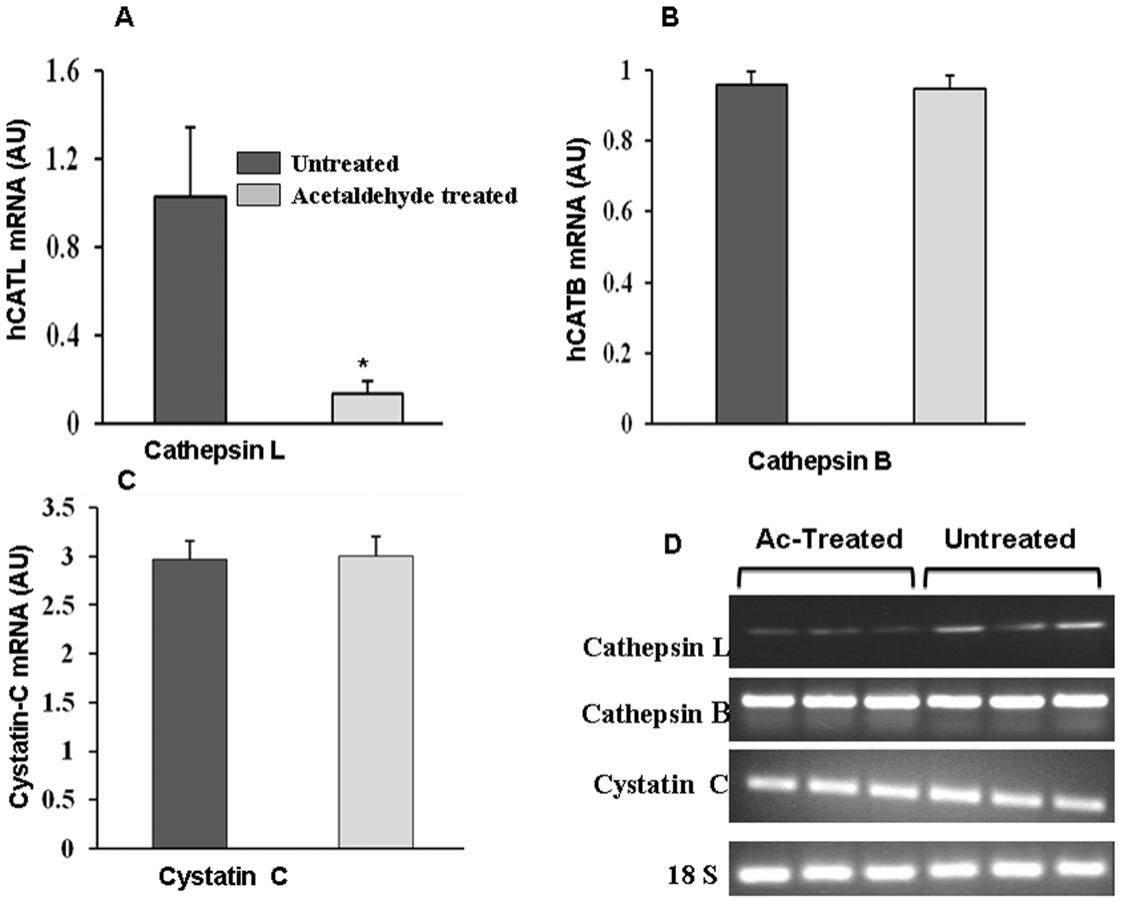
Reduction of CTSL mRNA in acetaldehyde treated HepG2 cells. Total cellular RNA isolated from acetaldehyde treated or untreated cells was reverse transcribed and subjected to real time PCR using sybergreen and gene specific primers for (A) CTSL (B) cathepsin B or (C) cystatin C. Similarly real time PCR for 18 S rRNA was performed and used as an internal control. Cycle threshold (Ct) values were calculated for each PCR and relative fold change was calculated using 2−^ΔΔ Ct^ method. Each set of observation was compared to the other set using a paired two-tailed t-test, assuming unequal variances among the sample means. A p value of ≤0.05 was considered to be statistically significant. Values are mean ± SD from three independent experiments performed in triplicates. Values significantly different from control has been marked by *. (D) Analysis of PCR products. After the completion of real time PCR amplified products were resolved on agarose gel to confirm the amplification of a single product. Representative gel for each target is given in the figure.

In the present study we used 5.0 µM concentration of acetaldehyde to study its effect on expression of CTSL. To assess the toxicity of acetaldehyde at this concentration we compared the growth rates of HepG2 cells in its presence and absence and the results are presented in figure 1. As evident from these results acetaldehyde treated and untreated cells exhibited similar growth rates and thus ruled out any cytotoxic effect of acetaldehyde on these cells at 5.0 µM concentration. However, treatment of HepG2 cells with this non-cytotoxic concentration of acetaldehyde lead to a significant reduction (p = 0.01; 2.3 fold) in the activity of CTSL assayed fluorimetrically using a synthetic substrate CBZ-Phe-Arg-Nmec (Figure 2A). However, no significant difference in the activity of cathepsin B was observed at this concentration of acetaldehyde (Figure 2B). To ascertain that the observed decrease in CTSL was not due the direct inhibition of the enzyme by acetaldehyde we quantiated this protease by Western blotting and a representative blot is presented in (Figure 2C). hCTSL is synthesized as a 42 kDa pre-proenzyme which is processed to a 34 kDa proenzyme and ultimately to a 26 kDa enzymatically active form. As evident from figure 1C all these forms were detected by the antibody used in the present study. Densitometry of these blots revealed a 2.5 fold reduction in all three forms of CTSL (Figure 2D). Since there was no effect on the levels of α-tubulin upon this treatment, we concluded that acetaldehyde down regulates the expression of CTSL. Consistent with these results we observed significantly lower levels of CTSL mRNA in acetaldehyde treated cells (Figure 3A). However, this treatment did not affect the levels of cathepsin B, cystatin C mRNA or 18S RNA (Figure 3B, C and D). These results suggest that acetaldehyde decrease CTSL expression either by decreasing its gene transcription or mRNA stability.

### Transcriptional down regulation of hCTSL expression by acetaldehyde

**Figure 4:**
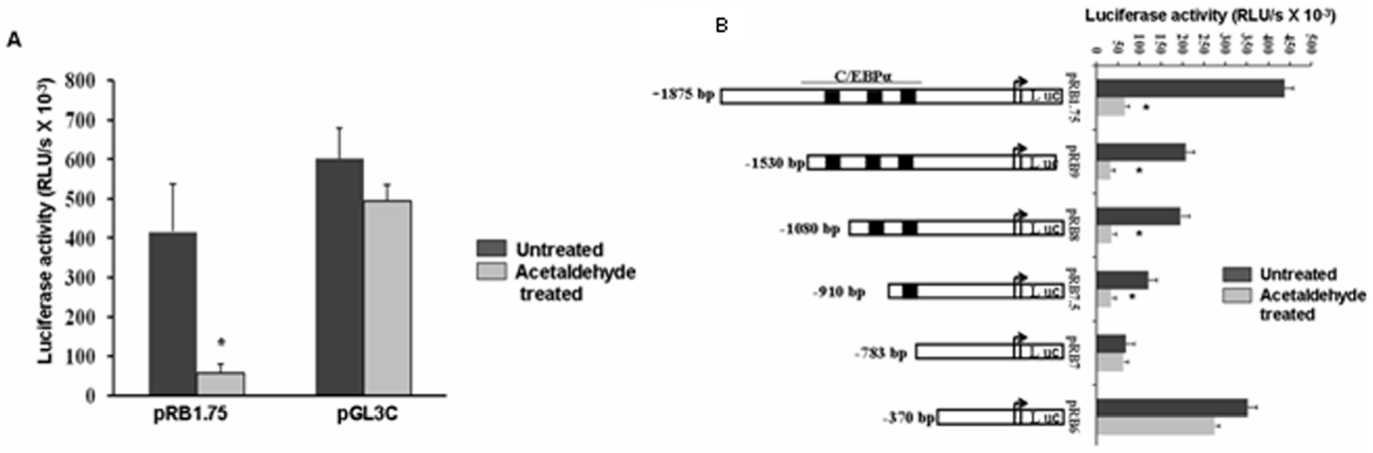
Effect of acetaldehyde on CTSL promoter activity and identification of acetaldehyde response element. (A). CTSL promoter activity in acetaldehyde treated and untreated cells. HepG2 cells were transfected with CTSL promoter reporter construct (pRB1.75) or pGL3C (control) vector. After 24 hours the transfected cells were treated with 5.0 µM acetaldehyde or vehicle solution (PBS) for another 24 hours and then the cells were lysed followed by assaying luciferase activity in the cell lysate. (B). Identification of acetaldehyde response elements in CTSL promoter by deletion analysis. HepG2 cells transfected with various CTSL promoter reporter deletion constructs were cultured in the presence or absence of acetaldehyde as described above and luciferase activity was assayed. All constructs were cotransfected with the pRLnull vector and the renilla luciferase activity was used to normalize for the transfection efficiency. Each transfection was done in triplicates and results are expressed as mean ± SD from three independent experiments. Values significantly different from control has been marked by *.

In a previous study our laboratory cloned hCTSL promoter and characterized it by making a series of promoter reporter constructs [21]. We used these constructs to assess the role of transcription in down regulation of CTSL by this oxidative metabolite of ethanol. Initially for this purpose a hCTSL full length promoter reporter construct (pRB1.75) was transiently transfected in HepG2 cells and the activity of luciferase reporter gene was assayed after treatment with acetaldehyde or vehicle solution (PBS). As shown in Fig. 4A, compared to PBS, acetaldehyde treatment decreased CTSL promoter activity by 7.0 fold, which was statistically significant (p = 0.02). However, acetaldehyde did not affect luciferase activity when these cells were transfected with pGL-3C (luciferase cloned under SV-40 promoter) vector. These results convincingly established the specificity of acetaldehyde for hCTSL promoter and the role of transcription in decreasing CTSL expression by this metabolite of ethanol.

### Localization of acetaldehyde response element(s) in hCTSL promoter by deletion analysis

In an effort to identify the transcription factor binding motif(s) in hCTSL promoter which confer responsiveness to acetaldehyde we transfected the above mentioned series of promoter reporter deletion constructs in HepG2cells and assayed luciferase activity after acetaldehyde treatment. As shown in Fig. 4B, cells transfected with the reporter construct containing the entire 1.87 kb promoter region exhibited a 7.0 fold decrease in luciferase activity upon acetaldehyde treatment. As reported earlier [21], gradual deletion of 1092 bp (pRB-1.75, pRB9, pRB8, pRB7.5 pRB7) from the upstream end of hCTSL promoter resulted in a corresponding decrease in the luciferase activity. However, HepG2 cells transfected with all these constructs except pRB-7 exhibited significant reduction in luciferase activity after acetaldehyde treatment. Interestingly pRB-6, another promoter reporter construct lacking 413 bp from the 5′end of hCTSL promoter in pRB-7 lead to an increase in the luciferase activity both in the presence and absence of acetaldehyde. While Bakhshi et al [21], attributed the increase in promoter activity due to the deletion of this region to the presence of potentially negative regulatory element(s) in the deleted bases, results of this study suggest the presence of acetaldehyde response motif(s) in the promoter region upstream to −783 bp.

### C/EBP-α motif(s) confer acetaldehyde responsiveness to hCTSL promoter

**Figure 5:**
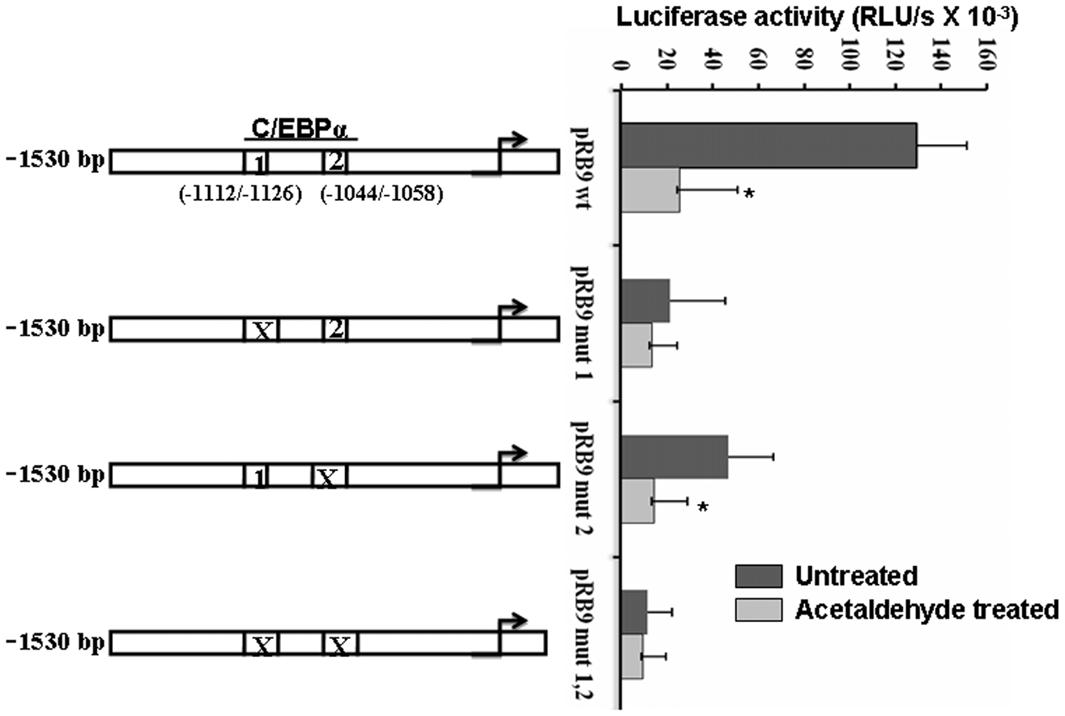
Mutagenesis of C/EBP α binding motifs abolishes CTSL promoter activity and its responsiveness to acetaldehyde. The two C/EBPα binding motifs were mutated individually or in combination and the resulting constructs were transfected in HepG2 cells. The mutated motifs have been marked by **X**. The luciferase activity of these constructs was assayed after culturing the transfected cells in the presence or absence of acetaldehyde for 24 hours. Values are mean ± S.D from three independent experiments. Values significantly different from untreated controls have been marked by *.

In view of the results of the previous experiment we analyzed the sequence of hCTSL promoter upstream to −783 for the presence of putative transcription factor binding motifs by an online tool http://.motif.genome.jp. This analysis besides several other transcription factor binding motifs reveled the presence of three putative C/EBPα binding sites at −1112/−1126; −1044/−1058. As deletion of the most upstream −1378/−1392 C/EBPα motif did not affect the promoter activity we explored the role of the other two sites in conferring acetaldehyde responsiveness to hCTSL promoter. For this purpose we mutated them sequentially as well as simultaneously. As shown in Fig. 5 mutagenesis of these two motifs (−1112/−1126; −1044/−1058) resulted in a significant decrease in (p = 0.01; 5.2 fold) the promoter activity and a total loss of responsiveness to acetaldehyde. A similarly decrease in promoter activity and acetaldehyde responsiveness was observed by mutagenesis of −1112/−1126 motif alone. However mutation of −1044/−1058 motif significantly reduced (p = 0.05) hCTSL promoter activity without affecting its responsiveness to acetaldehyde.

These results demonstrate that both C/EBPα binding motifs (−1112/−1126; −1044/−1058) are important for hCTSL promoter activity but −1112/−1126 motif is critical for its responsiveness to acetaldehyde.

### Binding of C/EBP α to its cognate motifs on hCTSL promoter

**Figure 6:**
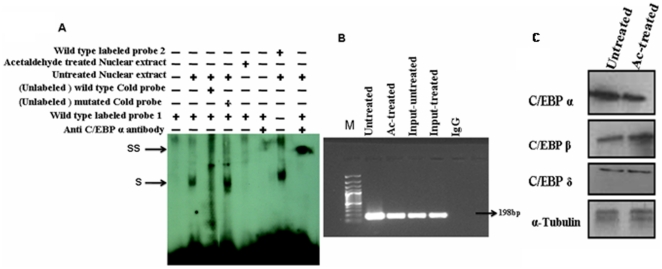
Acetaldehyde decrease/eliminates C/EBP alpha binding to CTSL promoter sequence. (A) Demonstration of specific binding of C/EBP α to CTSL promoter. Radio-labeled double stranded DNA fragment (14 bp) encompassing wild type or mutated C/EBP α binding motif 1 or 2 ( described in figure - ) were incubated with the nuclear lysate (5 µg protein) prepared from acetaldehyde treated or untreated cells in the binding assay buffer. The DNA protein complex were resolved on non denaturing 5% PAGE and detected by autoradiography. The binding reactions were also carried out in the presence of 100 molar excess unlabelled double stranded DNA containing wild type or mutant C/EBP α binding motif to ascertain the specificity of DNA protein interaction. In some of the reactions 10 µg of antibody against C/EBP α were incubated with or without nuclear lysate before adding radio-labeled probe. Shift and supershift in the protein DNA complexes have been marked by **S** and **SS** respectively. (B) In vivo binding of C/EBP-α to CTSL promoter. Crosslinked chromatin isolated from acetaldehyde treated or untreated HepG2 cells using C/EBP-α antibody were subjected to PCR with gene specific primers (nucleotide sequence given in table) flanking the C/EBP-α binding motifs on CTSL promoter. The amplified products were resolved on agarose gel. The cross linked chromatin immunoprecipitated with normal rabbit IgG were also subjected to PCR using the same primers and served as negative control. Amplification of an expected 198 bp fragment is evident in all the lanes except negative control. Input control is from non immunoprecipitated total genomic DNA. (C) Effect of acetaldehyde treatment on levels of C/EBP transcription factors. Cell lysates of acetaldehyde treated or untreated cells were resolved on SDS-PAGE and subjected to western bloting using specific antibodies for C/EBPα, C/EBPβ or C/EBPδ as described in Materials and Methods section. Simultaneously western blot for α-Tubulin was performed and used for normalization for equal loading.

EMSA were employed to assess the binding of C/EBPα to its cognate motifs essential for hCTSL promoter activity and conferring responsiveness to acetaldehyde. As shown in Fig. 6A incubation of nuclear lysate isolated from HepG2 cells (without treatment with acetaldehyde) retarded the mobility of double stranded radio-labeled DNA containing −1112/−1126 or −1044/−1058 C/EBP α binding motif suggesting the binding of nuclear proteins to these DNA fragments. This interaction of protein(s) to the radio-labeled probe was abolished by 100 molar excess of the same un-labeled DNA fragment (wild type C/EBP α motif) but not by the same unlabeled DNA containing mutated C/EBPα binding motif. These results demonstrated the specific binding of nuclear protein(s) to the CEBP α motif on radio-labeled probe. A further retardation in the mobility of the above mentioned protein - radio-labeled DNA complex by anti C/EBPα antibody confirmed that the bound nuclear factor to the radio-labeled probe was indeed C/EBPα.

However, no retardation in the mobility of the above mentioned radio-labeled probe was observed when it was incubated with the nuclear lysate isolated from acetaldehyde treated HepG2 cells. Thus based on these results it was concluded that acetaldehyde treatment abolished C/EBPα binding to its cognate motif on hCTSL promoter.

The *in vivo* binding of these motifs to the CEBP α was further confirmed by ChIp assays (Figure 6B). For these assays chromatin from the acetaldehyde treated and untreated HepG2 cells were immuno-precipitated by anti C/EBPα antibody. This chromatin was subjected to quantitative PCR using the primers flanking the above mentioned C/EBPα binding motifs. The nucleotide sequence of these primers is given in table-1. We observed amplification of the right size DNA fragments using chromatin obtained from both acetaldehyde treated and untreated cells. The identity of these fragments were confirmed by double stranded DNA sequencing (data not shown).however no DNA fragment of any size was amplified when chromatin immuno precipated using the total rabbit anti mouse IgG was used as template for PCR. Analysis of PCR products on agarose gel also revealed strikingly higher amplification of the target DNA fragment when chromatin immunoprecipitated from untreated HepG2 cells was used as template compared to when it was immunoprecipitated from acetaldehyde treated cells. Whereas input DNA from acetaldehyde treated and untreated cells exhibited comparable amplification of the target fragments. These results corroborated our EMSA results and confirmed that acetaldehyde reduces the binding of C/EBPα to its cognate motifs on hCTSL promoter. Interstingly, acetaldehyde reduced the C/EBPα expression without altering the levels of C/EBPδ. However C/EBPβ expression was increased by acetaldehyde treatment (Fig. 6C). Since hCTSL promoter contains two functional C/EBPα but no C/EBPβ or C/EBPδ binding motifs, our results suggested that reduction in C/EBPα levels in response to acetaldehyde treatment is responsible for decreased CTSL expression in HepG2 cells.

### Reduction in CTSL levels and its promoter activity by siRNA mediated silencing of C/EBPα

**Figure 7:**
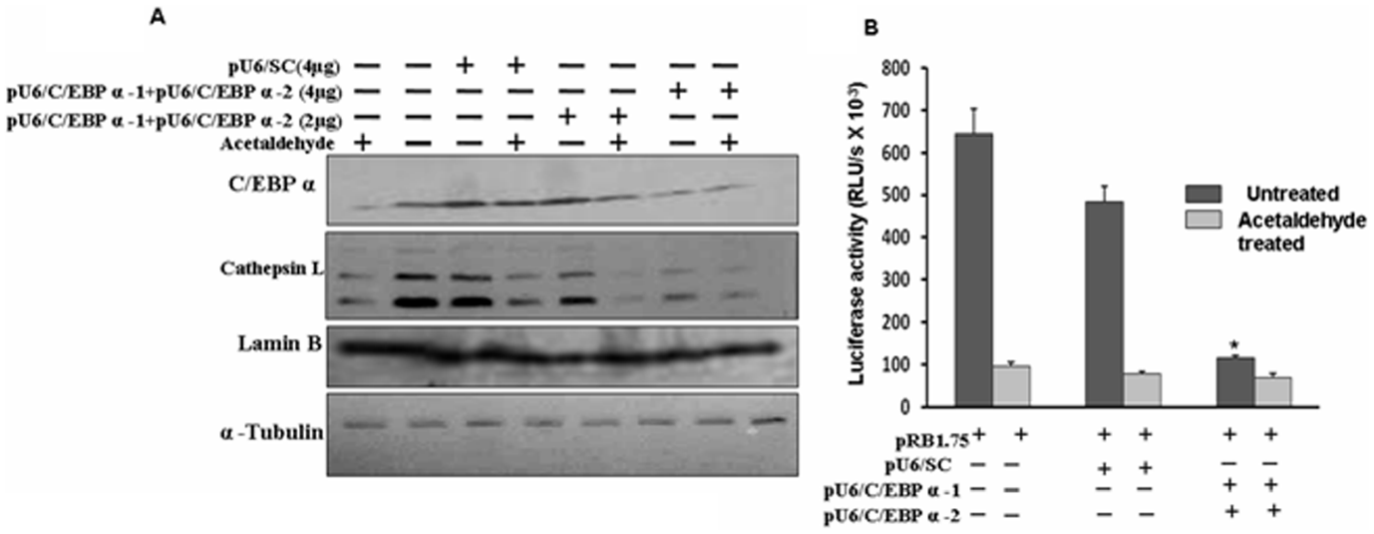
Silencing of C/EBP α expression simulate the effect of acetaldehyde. (A). Simulation of the effect of acetaldehyde treatment on CTSL expression by Silencing of C/EBP α expression. HepG2 cells transfected with 2.0 or 4.0 µg of C/EBP α si RNA expression vectors ( pU6-C/EBPα-1+pU6-C/EBPα-2 ) or 4.0 µg of scrambled Si RNA expression vector(pU6-SC) were cultured in the presence or absence of 5 um acetaldehyde as described in figure 3. After 24 hours the cells were lysed and levels of C/EBP α, CTSL, lamin B and α tubulin were detected by western blotting using specific antibodies. The cell lysates prepared from untransfected, untreated or treated with acetaldehyde cells were also subjected to western blotting using the above mentioned antibodies and served as control. Each blot shown is a representative of at least three separate experiments. (B). Silencing of C/EBP α expression simulates the effect of acetaldehyde treatment on CTSL promoter activity. HepG2 cells co transfected with 1.0 µg of pRB-1.75 and 4.0 µg of C/EBP α si RNA expression vectors ( pU6-C/EBPα-1+pU6-C/EBPα-2 ) or scrambled Si RNA expression vector(pU6-SC) were cultured in the presence or absence of 5 µM acetaldehyde as described in figure 3. After 24 hours the cells were lysed and luciferase activity was assayed. Each transfection was done in triplicates and results are expressed as mean ± SD from three independent experiments. Values significantly different from control has been marked by *.

Deletion analysis, site directed mutagenesis, EMSA and CHIP assay established the specific binding of C/EBPα to its cognate motifs on hCTSL promoter and its role in conferring acetaldehyde responsiveness. To confirm that C/EBPα alone was sufficient to mediate CTSL reduction by acetaldehyde we used C/EBPα siRNA. As shown in Fig. 7A transfection of C/EBPα siRNA expression vector decreased the expression of C/EBPα in HepG2 cells in dose dependent manner. This decrease was comparable to its level in acetaldehyde treated cells. However the expression of this transcription factor remained unaltered by the scrambled siRNA. Similarly the expression of lamin b and α-tubulin were not affected by C/EBPα siRNA confirming its specificity against C/EBPα alone. Consistent with the findings of mutagenesis experiments, reduction in C/EBPα levels was accompanied by a parallel decrease in the CTSL levels. Thus siRNA mediated silencing of C/EBPα could mimic the effect of acetaldehyde on CTSL expression in HepG2 cells. These results established beyond doubt that reduction in C/EBPα alone was sufficient to down regulate CTSL expression in these cells and thereby play a key role in conferring acetaldehyde responsiveness to hCTSL promoter.

Finally we used siRNA mediated silencing to confirm that the CEBP-α down regulation was sufficient to reduce CTSL promoter activity. As evident from Fig. 7 B co-transfection of pRB-1.75 with C/EBP-α siRNA expression vector in HepG-2 cells resulted in 80% reduction (p = 0.001) in hCTSL promoter activity. This reduction was barely 24% when it was co-transfected with scrambled siRNA vector. Acetaldehyde treatment could not cause any further reduction in the C/EBPα siRNA mediated decrease of hCTSL promoter activity. Whereas cells co-transfected with pRB-1.75 and scrambled siRNA vector exhibited 81% reduction (p = 0.03) in luciferase activity upon acetaldehyde treatment compared to the untreated control. Thus C/EBPα siRNA could decrease hCTSL promoter activity to its level in acetaldehyde treated cells. These results allowed us to conclude that silencing of C/EBPα alone is sufficient for transcriptional down regulation of CTSL expression.

### Reduction in CTSL and C/EBPα expression by acetaldehyde treatment in human hepatic stellate cells

**Figure 8:**
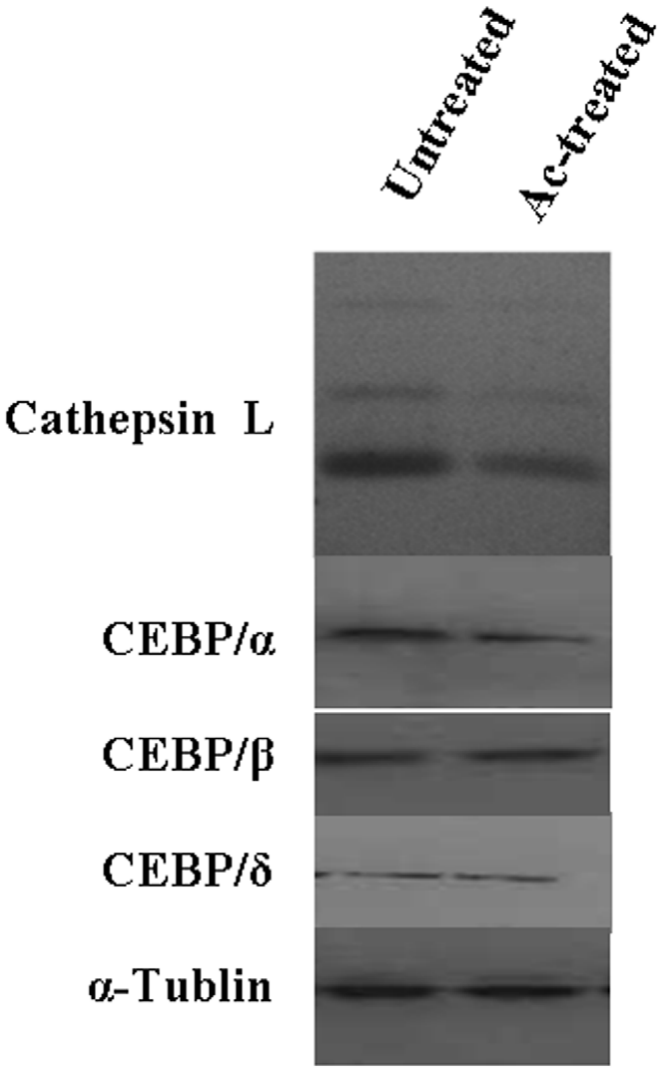
Acetaldehyde treatment decreases CTSL and C/EBP α level in human hepatic stellate cells. Cell lysates of acetaldehyde treated or untreated hepatic stellate cells were resolved on SDS-PAGE and subjected to western bloting using specific antibodies for CTSL, C/EBPα, C/EBPβ or C/EBPδ as described in Materials and Methods section. Simultaneously western blot for α-Tubulin was performed and used for normalization for equal loading.

Our results convincingly demonstrated C/EBP α mediated down regulation of CTSL by acetaldehyde in HepG2 cells. In view of their being major fibrogenic cells we also investigated the effect of acetaldehyde on the expression of CTSL and C/EBP isforms in hepatic stellate cells. Treatment of these cells with acetaldehyde (5 µM) did not exhibit any cytotoxic effect but resulted in a very noticeable decrease in CTSL expression (Fig. 8). A parallel reduction in the levels of C/EBPα was observed but no such difference in the expression of C/EBP β, C/EBP δ or α Tubulin was apparent after acetaldehyde treatment (Fig. 8). This parallel reduction in CTSL and C/EBPα expression by acetaldehyde taken together with the results of the previous experiments described in this study, suggest C/EBPα mediated down regulation of CTSL levels in both HepG2 and hepatic stellate cells with this metabolite of ethanol by a common mechanism.

## Discussion

Acetaldehyde, an oxidative metabolite of ethanol produces complex interplay of effects, including mitochondrial dysfunction, autoimmune mediated cell injury, oxidative stress and overproduction of inflammatory cytokines, which finally culminates in the development of the alcoholic liver diseases [23]–[26]. Most of the acetaldehyde induced liver injury occurs in the perivenous zone around hepatocytes [27]–[29], followed by the accumulation of ECM proteins such as collagens (type-1, III, IV, V and XVIII), elastin, laminin and fibronectin etc [30]. Acetaldehyde induces the synthesis of matrix proteins in hepatic stellate cells [31], [32] and inhibits hormone stimulated DNA synthesis in hepatocytes [33]. It has also been reported to modulate the expression of extracellular matrix degrading proteases like MMP-1, MMP-2 [7], uPA [34] and MMP-9 [8], [35] in human stellate cells and hepatocytes respectively. Since no such information on ECM degrading protease cathepsin L was available, primary objective of the present study was to investigate the effect of acetaldehyde on expression and activity of this protease.

Our results demonstrate reduction in the levels of immuno reactive CTSL in hepatocytes and hepatic stellate cells treateted with noncytotoxic concentrations of acetaldehyde. Use of HepG2 cells to elucidate the molecular mechanism of this reduction revealed a parallel decrease in the levels of transcription factor C/EBPα and expression and promoter activity of this protease suggesting it's transcriptional down regulation by acetaldehyde. Hepatic stellate cells after acetaldehyde treatment exhibited a similar decrease in C/EBPα and CTSL levels. Consistent with our findings Findik et al [36] also observed significant reduction in C/EBPα protein in ethanol treated mouse liver and VL17 A cells. In the present study we demonstrate that SiRNA mediated silencing of C/EBPα can mimic the effect of acetaldehyde on CTSL expression. Similarly mutagenesis of C/EBPα binding motifs can abolish responsiveness of hCTSL promoter to acetaldehyde. These results led to us to conclude the key role of C/EBPα in mediating downregulation of CTSL expression by acetaldehyde. Similar observations were made by Findik et al [36], in case of hepcidin promoter in HepG2 cells.

Acetaldehyde induces collagen α1 (I) expression with the involvement of C/EBP-β family of transcription factors with a mechanism that requires H_2_O_2_ generation, an insight that directly relates oxidative stress and acetaldehyde induced collagen synthesis [37]. Similarly Attard et al, [38], observed increased expression and promoter binding of C/EBP-β leading to enhanced collagen α1 (I) synthesis in response to acetaldehyde. In the present study HepG2 cells also exhibited increased expression of C/EBPβ after acetaldehyde treatment. However, the expression of C/EBPδ remained unaffected by this treatment.

Over expression of CTSL leads to its secretion in to the extracellular milieu [13]. Also the localization of CTSL on the hepatocytic villi is documented [39]. Since it is very potent in degrading collagen, elastin, laminin, fibronectins and other components of ECM CTSL present on hepatocytic membrane and in neighboring milieu along with other proteases may be expected to control the turnover of extra cellular matrix. Interestingly, ethanol administration significantly decreased the severity of destructive arthritis characterized by collagen degradation in mice [40]. A specific ribozyme of CTSL exhibited a similar reduction in the severity of destructive rheumatoid arthritis thereby confirming the involvement of this protease in collagen and proteoglycan degradation [41]. This is consistent with the observation that blocking the secretion of CTSL can abolish metastasis of melanoma cells, a process which requires ECM degradation [14]. In view of these reports, results of the present study, suggest that acetaldehyde mediated reduction in CTSL expression may at least partly contribute to the accumulation of matrix proteins in alcoholic liver fibrosis. Elevated expression of matrix proteins in response to acetaldehyde by hepatic stellate cells may further contribute to this process [31], [32].

Pathological angiogenesis, irrespective of the etiology has been extensively described in chronic liver diseases [42]. It is governed by a delicate balance between pro and anti angiogenic factors. Endostatin a very potent anti-angiogenic factor is generated from collagen XVIII by secreted CTSL [43]. A decrease in the synthesis, secretion and activity of CTSL will result in the decreased endostatin production which in turn will favor angiogenesis. Therefore, reduction in CTSL levels by acetaldehyde observed in the present study may explain the formation of new vessels that are closely associated with the pattern of fibrosis development typical of alcoholic liver disease [42]. However, the role of decreased CTSL expression in inducing angiogenesis during liver fibrosis is yet to be established experimentally.

In summary results of the present study demonstrate C/EBPα mediated down regulation of the CTSL expression by acetaldehyde in HepG2 and hepatic stellate cells. This reduction in view of its ability to efficiently degrade ECM proteins may at least partly be responsible for shifting of balance of these proteins in favor of their deposition in the liver.
